# High-Flow Nasal Oxygenation During Sedation for Transcatheter Aortic Valve Replacement: The HIGH-OXY-TAVR Randomised–Controlled Trial

**DOI:** 10.3390/jcm14238347

**Published:** 2025-11-24

**Authors:** Marc Giménez-Milà, Antoni Manzano-Valls, Omar Abdul-Jawad, María José Arguis, Salvatore Brugaletta, Thiago Carnaval, Maria José Carretero, Eduardo Flores-Umanzor, Xavier Freixa, Cristina Ibañez, Stefano Italiano, Manuel López-Baamonde, Samira Martínez-Otero, Purificación Matute, Mireia Pozo, Ricard Navarro-Ripoll, Juan Manuel Perdomo, Ander Regueiro, Irene Rovira, Francisco Javier Vega, Sebastián Videla, Manel Sabaté

**Affiliations:** 1 Department of Anaesthesia and Intensive Care, Hospital Clinic of Barcelona, 08036 Barcelona, Spain; magimene@clinic.cat (M.G.-M.); mpozoal@clinic.cat (M.P.);; 2 Institut d’Investigació August Pi I Sunyer (IDIBAPS), 08036 Barcelona, Spain; 3 Facultat de Medicina i Ciències de La Salut, Universitat de Barcelona (UB), c. Casanova, 143, 08036 Barcelona, Spain; 4 Cardiology Department, Hospital Clínic, Universitat de Barcelona, 08036 Barcelona, Spain; 5 Pharmacology Unit, Department of Pathology and Experimental Therapeutics, School of Medicine and Health Sciences, IDIBELL, University of Barcelona, 08907 L’Hospitalet de Llobregat, Spain; 6 Clinical Research Support Area, Clinical Pharmacology Department, Hospital Universitari Germans Trias i Pujol, 08916 Badalona, Spain; 7 Department of Pharmacology, Therapeutics and Toxicology, Autonomous University of Barcelona, 08193 Bellaterra, Spain; 8 Centro de Investigación Biomédica en Red de Enfermedades Cardiovasculares (CIBERCV), 28029 Madrid, Spain

**Keywords:** aortic stenosis, high-flow nasal oxygenation, hypoxia, sedation, transcatheter aortic valve replacement

## Abstract

**Background:** Data on high flow nasal oxygenation (HFNO) efficacy in hypoxia prevention in transcatheter aortic valve replacement (TAVR) are conflictive. We aimed to determine the benefit of HFNO in preventing the occurrence of desaturations during TAVR. **Methods:** An investigator-initiated, proof of concept, single-centre, randomised, and controlled trial on 132 adult patients who were scheduled to undergo transfemoral TAVR was conducted. Patients were randomised (1:1) to HFNO (H-group) with a flow rate of 50 L min^−1^ and FiO_2_ 0.6 or standard of care oxygen therapy (S-group). The primary endpoint was the number of patients with a desaturation episode (SpO_2_ < 93%) for >10 s during TAVR. Secondary outcomes included arterial partial pressure of oxygen (pO_2_) 45 min from sedation start and changes in glomerular filtration rate from baseline to 12 h post-procedure. **Results:** Between 23 November and 24 July, a per-protocol analysis was performed in a total of 125 patients (H-group *n* = 64; S- group *n* = 61; 49 females). The number of patients with any desaturation episode was significantly lower in the H-group [13/64 (20%, 95% CI: 12–32%)] than in the S-group [31/61 (51%, 95% CI: 39–63%), RR: 0.39 (95%CI: 0.23–0.68)]. At 45 min, mean (SD) pO_2_ was higher in the H-group (24(9.8) kPa vs. 16.7(7.5) kPa; *p* < 0.005). A significant improvement in delta median (IQR) difference on glomerular filtration rate was observed in the H-group [1.6(−1–7.9) mL min^−1^ 1.73 m^−2^] with respect to the S-group [0.2(−6.1–3.1) mL min^−1^ 1.73 m^−2^; *p*-value: 0.013]. **Conclusions:** This trial demonstrated that HFNO provides a better oxygenation pattern than standard oxygen therapy during TAVR. Larger studies focusing on long-term clinical outcomes are warranted to evaluate the benefit of HFNO during sedation for TAVR procedures.

## 1. Introduction

The anaesthetic approach for transcatheter aortic valve replacement (TAVR) procedures has evolved in recent years, from general anaesthesia techniques to a less invasive strategy such as sedation with variable anaesthetic plans: superficial/conscious and deep depending on patient status. Currently, sedation represents the standard of care in the majority of cases [1]. In a few observational studies, sedation, compared to general anaesthesia, showed better haemodynamic stability and shorter in-hospital and intensive care unit stays [2,3]. It is also associated with a lower postoperative recovery time, which eases patient management and facilitates patient transfer from the cathlab to the ward or home in ambulatory TAVR programmes, improving the overall procedure efficiency.

However, sedation may lead to respiratory depression, apnoea, and airway obstruction with the development of hypoxia and hypercapnia. This can further exacerbate pulmonary hypertension and contribute to a systemic vasodilation [4,5]. Moreover, patients undergoing TAVR procedures are at a higher risk of hypoxia, due to pre-existing patient-related factors such as obesity, obstructive sleep apnoea, chronic heart/lung disease, and renal failure [6,7].

To prevent the development of hypoxia, supplemental oxygen is traditionally provided during sedation via nasal cannulas or face masks [8]. Non-humidified nasal oxygen cannot exceed 2–5 L min^−1^ without causing damage to the nasal mucosa, and the percentage of oxygen delivered via variable-flow face masks is difficult to predict when administered rendering an inexact oxygen concentration (FiO_2_) [9,10].

High-flow nasal oxygen therapy (HFNO) was developed to provide flow rates of up to 70 L min^−1^ through specifically adapted nasal cannulas. It reliably delivers FiO_2_ between 0.21 and 1 [11,12]. Randomised–controlled trials have studied the effect of HFNO in sedation for gastro-intestinal and dental procedures showing an overall benefit compared to conventional dry nasal cannula in reducing the hypoxic risk [13,14,15].

Patients undergoing TAVR represent a high-risk population of respiratory complications during procedural sedation, which could simultaneously impact their inherent compromised hemodynamic condition of severe aortic stenosis. Nevertheless, a UK-based randomised–controlled trial that involved 73 individuals undergoing TAVR failed to show an improvement in gas exchange with HFNO at 50 L min^−1^ FiO_2_ 0.3 measured by arterial pO_2_ and pCO_2_ [16]. However, fewer desaturation episodes (secondary endpoint) were observed with HFNO.

The aim of this trial was to determine the benefit of HFNO in preventing the occurrence of episodes of hypoxemia compared to the use of nasal cannula during sedation. Secondarily, as hypothesis generating, we assessed the impact of HFNO on changes in specific biomarkers such as creatinine, N-terminal pro-B-type natriuretic peptide (NT-proBNP), and high-sensitivity cardiac troponin I (hs-cTnI).

## 2. Methods

### 2.1. Trial Design

This study was an investigator-initiated, proof of concept, single-centre, open, randomised, and controlled trial involving adult patients scheduled to undergo transfemoral TAVR procedures under sedation. It was carried out in strict compliance with the Declaration of Helsinki (version during trial execution; Fortaleza, Brazil, October 2013) and conducted following the legal requirements established under the European Regulation 2017/745 and Royal Decree 192/2023 on Medical Devices. All patients were provided with written informed consent before their inclusion. The ethics committee approved the trial protocol on 19 May 2023, registered as HCB/2022/1095 at Hospital Clinic de Barcelona, Spain. Data were treated confidentially, and CONSORT guidelines for conducting randomised–controlled trials were followed. De-identified data can be accessed upon request to the investigators.

### 2.2. Study Population

All patients who were scheduled to undergo TAVR procedures at our centre were screened for eligibility. Patients were eligible if they were 18 years of age or older and scheduled to undergo elective TAVR via transfemoral access. The main exclusion criteria included patient refusal to participate, non-transfemoral TAVR, procedure duration < 45 min, planned general anaesthesia due to clinical or technical reasons, or the need for general anaesthesia conversion due to procedural complications.

### 2.3. Randomisation and Blinding

Before the TAVR procedure, eligible patients were randomly assigned to receive HFNO or supplemental oxygen via nasal cannula, with the latter being the standard of care of oxygen therapy supply. Randomisation was performed by a system of blocks comprising between 4 and 6 patients with the use of computer-generated sequence. A web-based central randomisation service (Blockrand 1.5 version of R programme) ensured the concealment of trial-group assignment up to the beginning of the TAVR procedure. Due to the nature of the intervention, patients, physicians, and nurses were not blinded to the assigned group. However, the research personnel collecting the data, including the number of desaturation episodes and blood gas measurements, were blinded. Recruiters did not have access to the sequence randomisation list.

### 2.4. Trial Intervention

Baseline (<24 h prior TAVR implantation) measurements of serum creatinine, neuronal specific enolase (NSE), hs-cTnI, and NT-proBNP were performed in the biochemical laboratory of the study centre, with all reagents being CE-IVD marked. At the time of intervention, standard patient monitoring included a pulse oximeter, electrocardiogram and radial invasive pressure. This was inserted prior to commencing sedation and was used for sampling blood determinations of arterial pO_2_ and pCO_2_ with a point-of care analyser (RapidPoint 500 analyser, Siemens Healthineers, Forchheim, Germany).

Sedation was performed by the anaesthesiology team with a pre-programmed target-controlled infusion of propofol and remifentanil with the pharmacokinetics model of Schnider and Minto, respectively, aiming to achieve a Richmond Agitation Sedation Scale (RASS) of −4 to −2 prior TAVR procedure commenced. No other sedative premedication was administered to patients. A second measurement of arterial pO_2_ and pCO_2_ was performed at 45 min from the sedation start in same the point-of care analyser as the baseline sample. After the procedure, patients stayed in the post-anaesthesia recovery unit until they were ready for discharge to the ward. Another blood sample was obtained at 8–12 h to measure the serum creatinine, NSE, hs-cTnI, and NT-proBNP in the biochemical laboratory study centre.

Supplemental oxygen administration started at the same time as the sedative infusion. Patients in the intervention group (H-g) were administered warm and humidified oxygen at 50 L min^−1^ with 0.6 FiO_2_ via the Optiflow THRIVE Nasal High Flow delivery system (Fisher and Paykel, Auckland, New Zealand) as previously reported [16]. In the standard of care oxygen group (S-g), supplemental oxygen was provided using conventional dry nasal cannula at a flow rate of 5 L min^−1^.

The TAVR procedure was performed using a transfemoral approach and percutaneous ultrasound-guided femoral artery access. Both balloon-expandable and self-expanding valves could be implanted. These included Edwards SAPIEN 3^™^ (Edwards Lifesciences, Irvine, CA, USA), Myval^™^ prosthetic valve (Meril Life Sciences Pvt Ltd., Vapi, India), Medtronic Evolut PRO+ and FX^™^ (Medtronic, Minneapolis, MN, USA), Navitor^™^ TAVI systems (Abbott Cardiovascular, Abbott Park, IL, USA), and Acurate Neo2^™^ TAVI (Boston Scientific, Marlborough, MA, USA). Right ventricular pacing with a transvenous temporary pacemaker or left ventricular pacing using the pre-shaped wire was performed based on the operator’s preference. Sodium heparin (1 mg kg^−1^) was administered after vascular access was secured, and further doses were administered aiming for an activated coagulation time of >250 s.

### 2.5. Endpoints

The primary endpoint was the number of patients with a desaturation episode during the TAVR procedure. A desaturation episode was defined as SpO_2_ < 93% for more than 10 s [16].

Secondary endpoints included the total number of desaturation episodes during the TAVR procedure; the number of patients requiring manual ventilation due to respiratory depression; changes in arterial pO_2_ and pCO_2_ from baseline to 45 min after the start of sedation; and changes in end-organ damage biomarkers, including creatinine, NSE, hs-CTnI, and NT-proBNP, measured before the procedure and 12 h afterwards. The glomerular filtration rate was calculated with the CKD-EPI formula. The 45 min time period was proposed to allow a minimum time frame to evidence changes in respiratory function bearing in mind the average time of a TAVR is around 1 h. Acute kidney injury was defined according to VARC 3 definitions, as measured by serum creatinine [17].

### 2.6. Statistical Analysis

The sample size was calculated assuming an incidence of 30% of patients with at least 1 desaturation episode during the TAVR procedure in the S-g versus 10% in the H-g [16], with an 80% power and a two-tailed significance level of 5%. These assumptions lead to a sample size of 124 patients (62 per group). However, considering a potential 5% dropout rate, the final sample size was set at 132 individuals. No interim analysis was planned.

Categorical variables are presented as frequencies and percentages, and comparisons between groups were performed using the Pearson’s χ^2^ test with Yates’ continuity correction, as appropriate. Continuous variables are summarised as the mean (SD) or median (inter-quartile range [IQR]), depending on data distribution. The Shapiro–Wilk test was used to assess the normality of continuous variables, and distributional assumptions were further evaluated using Q-Q plots and goodness-of-fit analyses (Akaike Information Criterion, AIC). Delta analyses were performed to assess changes in physiological and biochemical parameters between baseline and postoperative values. The selection of statistical tests for delta values (Student’s *t*-test or Wilcoxon rank-sum test) was based on the distribution of the calculated changes. To evaluate the precise value of HFNO vs. standard of care, a per-protocol analysis was performed, with the primary analysis in the per-protocol population, which is defined as all randomised patients who received the assigned treatment. In addition, an intention to treat analysis was performed, including all patients randomised regardless of the treatment received.

Univariate logistic regression analyses were conducted to identify potential predictors of desaturation events (binary outcome). Odds ratios (ORs) with 95% confidence intervals (CIs) were calculated. To explore varying levels of risk, different thresholds for predictors were tested, ensuring a robust evaluation of their clinical significance. Significant predictors identified in univariate analyses were included in a multivariate logistic regression model to evaluate their independent effects, adjusting for potential confounders and interactions.

All statistical tests were two-sided, with a significance level set at *p* < 0.05. Statistical analyses were performed using R software version 4.2.3 (R Foundation for Statistical Computing, Vienna, Austria) for macOS^®^.

## 3. Results

### 3.1. Baseline Characteristics

**Figure 1:**
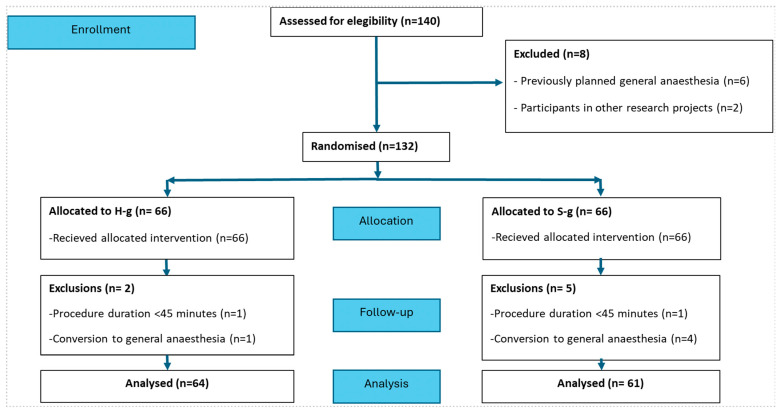
Study flow-chart with a total 125 patients in the per-protocol analysis.

Between November 2023 and July 2024, a total of 140 patients were screened for eligibility (Figure 1). Two patients were excluded due to participation in other research projects and six for an initially planned general anaesthesia approach. Thus, a total of 132 patients were deemed eligible for participation in the study and were enrolled after signing the informed consent of the study and randomised to H-g (*n* = 66, 50%) or S-g (*n* = 66, 50%). In the per-protocol analysis, five patients were excluded after randomisation due to crossover to general anaesthesia to allow vascular surgical repair (1 H-g, 4 S-g) and two patients (1 H-g, 1 S-g) due to procedure duration < 45 min. No patient in S-g required crossover to HFNO therapy. Therefore, in the per-protocol analysis, the final population of the study comprised a total of 125 patients with 64 allocated to the H-g (51%) and 61 to S-g (49%). The demographics, clinical characteristics at baseline, and procedural variables were similar in both groups (Table 1 and Table 2). Patients were mostly in the advanced NYHA class (65% in class III; 5% in class IV), with no differences between groups. Most common comorbidities included hypertension, dyslipidaemia, and chronic kidney disease. The median (IQR) baseline pO_2_ was 10.5 (9.6–11.7) kPa in S-g and 10.8 (9.7–11.6) kPa in H-g. Two patients from the H-g had a baseline pO_2_ < 7.9 kPa, being classified as pre-procedure hypoxemic respiratory failure, while none was observed in the S-g. There were no patients with baseline hypercapnia in either group. Overall, the mean left ventricle ejection fraction was preserved, and the pre-procedural mean aortic gradient was similar between groups (45 mmHg in H-g vs. 45 mmHg in S-g).

### 3.2. Procedural Characteristics

Self-expanding valves were mostly used in both groups (Table 2), with Evolut^™^ being the most frequently valve implanted. Rates of pre-dilatation and post-dilatation were similar in both groups. The median [IQR] dose of propofol was significantly higher in the H-g (168 mg [142–195] versus 144 mg [120–180] in S-g, *p*-value:0.006). The median [IQR] dose of remifentanil was similar in both groups (128 µg [118–167] in H-g versus 122 µg [88–157] in S-g, *p*-value: 0.18). The median [IQR] length of hospital stay was not significantly different between groups (4 days [1–28] in H-g versus 5 days [2–85] in S-g, *p*-value: 0.08).

### 3.3. Endpoints

**Figure 2:**
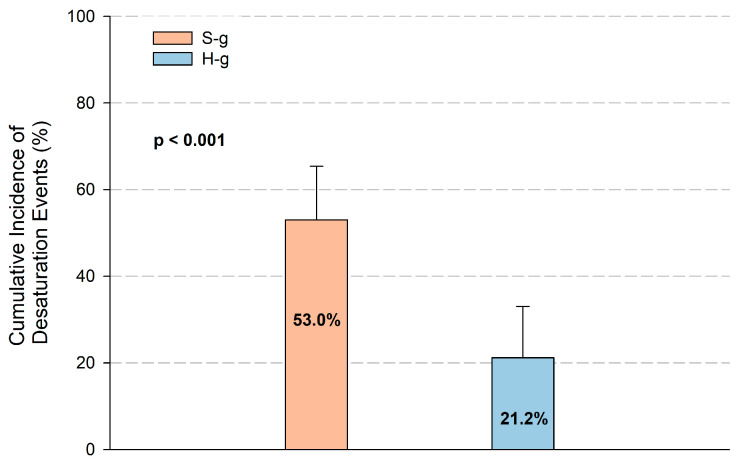
Cumulative incidence of at least one desaturation event in intervention and control groups. Number of patients that developed at least one episode of desaturation according to intention to treat analysis in H and S groups. The incidence was significantly higher in the S-g (53%) compared with the H-g (21%; *p* < 0.001). Error bars represent 95% confidence intervals.

The number of patients with at least one desaturation episode (primary endpoint) was statistically significant lower in the H-g compared to the S-g. In the intention to treat analysis, 21% (14 out of 66, 95% CI: 12–33%) of H-g patients experienced desaturation event vs. 53% (35 out of 66, 95% CI: 40% to 65%) of S-g patients (*p* < 0.001), RR: 0.4 (95% CI: 0.24–0.67). (Figure 2) [120–180].

According to the per-protocol analysis, the primary endpoint was significantly lower in the H-g [13 out of 64 (20%, 95% CI:12–32%) vs. 31 out of 61 (51%, 95% CI: 39–63%) in S-g; *p*-value: <0.001], RR: 0.39 (95% CI: 0.23–0.68).

Similarly, the median [IQR] number of desaturation episodes was statistically significantly lower in the H-g (0 [0–0] vs. 1 [0–3] in the S-g; *p*-value: <0.001. The number (%) of patients who needed manual ventilation due to respiratory depression was similar between groups [1(2%), 95% CI: 0–8% in the H-g vs. 3 (5%), 95% CI: 2–14%) in the S-g, *p*-value: 0.577]. At 45 min, the mean (sd) pO_2_ was significantly higher in the H-g (24 (9.8) kPa vs. 16.7 (7.5) kPa; *p*-value: <0.005), due to a statistically significant higher increase from the median (IQR) baseline pO_2_ (10.91 kPa (4.92–21.25) in the H-g vs. 4.07 kPa (1.45–8.76) in the S-g; *p*-value:0.001). The mean (sd) pCO_2_ values at 45 min, were comparable between groups (6.42 (1.37) kPa vs. 6.21(1.51) kPa; *p*-value: 0.390), Supplementary Figures S1 and S2.

### 3.4. End-Organ Damage Biomarkers

**Figure 3:**
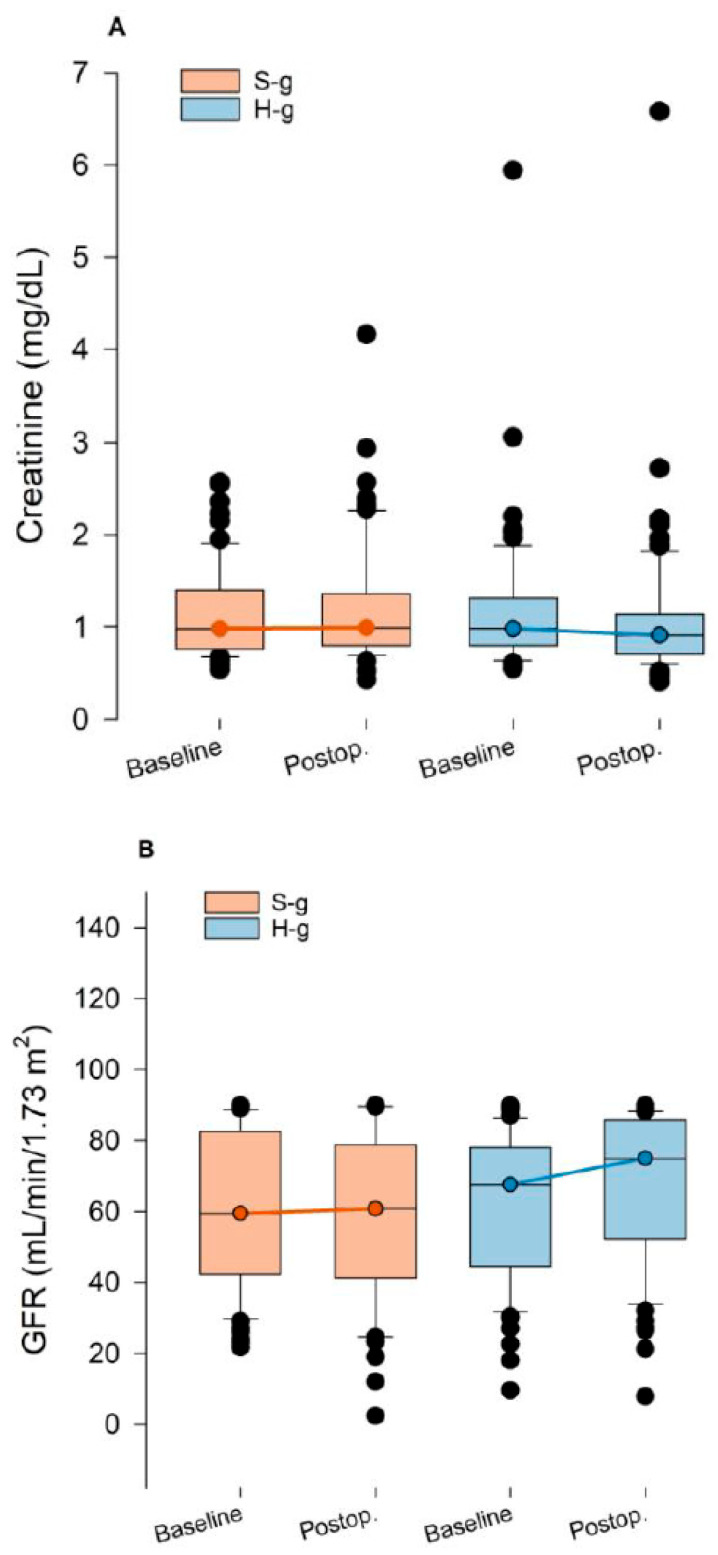
Trends in serum creatinine (**A**) and glomerular filtration rate (**B**) in H and S groups. Boxplots show median, interquartile range, and outliers for the S-g (orange) and H-g (blue) at baseline and postoperative time points. (**A**) Serum creatinine (mg dL^−1^). (**B**) Estimated glomerular filtration rate (GFR, mL min^−1^ 1.73 m^−2^).

Changes in end-organ damage biomarkers (8–12 h post-procedure vs. baseline values) are presented in Table 3 and Table S1, which contain both the intention to treat and per-protocol analyses. The glomerular filtration rate (mL min^−1^ 1.73 m^−2^) and creatinine (mg dL^−1^) were obtained in all subjects prior to and after TAVR. The median delta (IQR) creatinine levels decreased with statistical significance in the H-g −0.04 (−0.19 to 0.02) mg dL^−1^ vs. −0.01 (−0.11 to 0.13) mg dL^−1^ in the S-g; *p* = 0.020). In parallel, the median delta (IQR) glomerular filtration rate significantly increased in the H-g (1.6 (−1–7.9) mL min^−1^ 1.73 m^−2^ vs. 0.2 (−6.06 to 3.06) mL min^−1^ 1.73 m^−2^ in the S-g; *p*-value: 0.013). Figure 3 AKI was significantly less frequent in H-g compared to S-g (1 (stage-1) out of 64 (2%, 95%CI: 0–8%) compared to 7 (2 stage-1, 5 stage-2) out of 61 (12%, 95%CI: 6–22%) *p*-value: 0.024).

The median (IQR) NT-proBNP increased in the S-g 90.0 (−231.3–616.5) pg/mL^−1^ and in the H-g (55.0 (−204–208) pg mL^−1^). However, these differences were not statistically significant (*p*-value: 0.280). Changes in NSE and hs-cTnI did not differ between groups, see Supplementary Figures S3–S5.

### 3.5. Predictors of Desaturation Episodes

**Figure 4:**
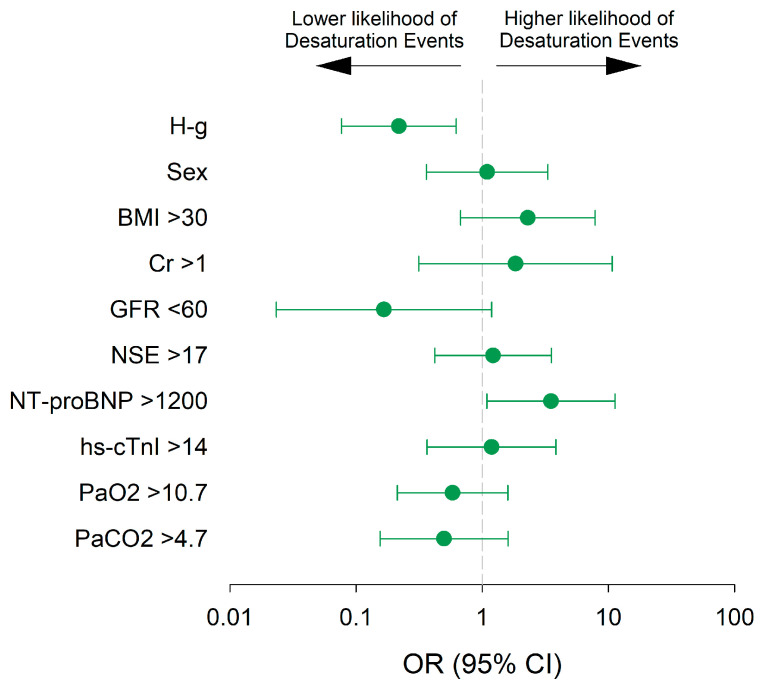
Multivariate regression analysis in the per-protocol population. Odds ratios (ORs) with 95% confidence intervals (CIs) are shown. Assignment to the H-g (vs. S-g) was independently associated with a significantly lower likelihood of desaturation events, while pre-procedural elevated NT-proBNP (≥1200 pg mL^−1^) was associated with increased risk. Other covariates (sex, age, arterial oxygen pressure [pO_2_], and atrial fibrillation) were not significantly associated with desaturation risk.

The results of the univariate and multivariate logistic regression analyses are presented in Figure 4 and Figure S6 and Supplementary Table S2. HFNO appeared to be independently associated with a protective effect on desaturations (OR 0.22; 95% CI 0.08, 0.63; *p*-value: 0.005), while a baseline NT-proBNP value > 1200 pg mL^−1^ was associated with an increased likelihood of desaturation (OR 3.50; 95% CI 1.09, 11.3; *p*-value: 0.035).

### 3.6. Safety and Mortality

No adverse events were attributed to HFNO or standard oxygen therapy. The in-hospital mortality and 30-day mortality was nil.

## 4. Discussion

This trial compares HFNO with conventional nasal cannula in patients undergoing TAVR requiring sedation. The results of this trial can be summarised as follows: (1) HFNO reduced the incidence and number of desaturation episodes in subjects undergoing TAVR; (2) the pO_2_ was improved by HFNO throughout the procedure; (3) HFNO was identified as an independent predictor against desaturation, while a baseline NT-proBNP > 1200 pg mL^−1^ was an independent predictor for desaturation, and (4) HFNO showed a significant improvement in renal function measured by the glomerular filtration rate.

The reduction in the number of desaturation episodes and the increased arterial pO_2_ during the procedure were observed in the H-g despite receiving a higher dose of propofol, with no differences in remifentanil as the short-acting opioid. The improved oxygenation profile obtained by HFNO probably allowed the anaesthesiologist to increase the propofol dose as a hypnotic drug to avoid patient movements that could potentially compromise the safety of the TAVR procedure. Of interest, there was no significant effect in the variation of pCO_2_ between groups. The relatively short duration of the procedure, combined with the higher doses of propofol received in the H-g, might explain the absence of differences in pCO_2_.

Herein, we have been able to show both a benefit in oxygenation measured with pO_2_ and to replicate the reduction in desaturation episodes [16]. The use of higher FiO_2_ (0.6 compared to 0.3) and a larger sample size may explain the differences between the studies.

HFNO therapy is a well-established respiratory support system with growing evidence in interventional gastro-intestinal and respiratory suites and intensive care medicine. However, the evidence in structural heart interventions is still limited, as TAVR patients were under-represented in previous HFNO trials [18,19,20,21].

The impact of advanced aortic valve disease added to patient frailty and comorbidities make the sedation of patients undergoing TAVR at extreme risk of periprocedural complications including haemodynamic collapse. Clinicians may face patients with advanced aortic stenosis and reduced left ventricle function who suffer from respiratory failure secondary to pulmonary oedema. An increased baseline NT-proBNP, a surrogate of heart failure, was identified as an independent predictor of desaturation. Thus, providing sedation to heart failure patients is a real challenge that can lead to respiratory depression, requiring conversion to general anaesthesia and invasive mechanical ventilation.

Increasing the oxygen concentration is a strategy used in critically ill patients. On the one hand, it is of paramount importance in clinical contexts of haemoglobin loss (i.e., acute haemorrhage) or of an increase in consumption (i.e., septic shock). Further, it is of potential benefit in reducing the risk of wound infection during surgical procedures. As a matter of fact, the World Health Organisation and Center for Disease Control have recommended the routine use of high inspired oxygen concentrations (FiO_2_ 0.8) during surgery and, if possible, several hours thereafter to reduce surgical site infection [22]. However, this recommendation has been extensively criticised, as the results of several meta-analyses failed to demonstrate a clear benefit of this strategy [23,24]. In the current study, we also aimed to evaluate the effects of HFNO on the prevention of end-organ damage during TAVR, a procedure that may carry haemodynamic and respiratory disturbances with consequences to vital organs (brain, kidney, heart). We observed an improvement in the acute renal function parameters in patients treated with HFNO after 12 h post-TAVR, with an uncertain impact on longer term renal function. Probably, improved systemic oxygenation in HFNO group led to a synchronic better renal oxygenation [25].

The link between renal hypoxia and development of AKI has been previously described in humans, with an increased renal oxygen extraction ratio observed in patients with AKI undergoing cardiac surgery [26,27]. Moreover, the development of AKI is a strong predictor of mortality both in patients undergoing TAVR with pre-existing chronic kidney disease and in those without [28,29,30]. The relationship between renal hypoxia and the development of AKI, as well as chronic kidney disease, has been previously described [25]. Thus, efforts to minimise this risk of AKI are of clinical interest.

Pre-existing heart failure (i.e., NT-proBNP > 1200 pg mL^−1^) was identified as an independent predictor of desaturation in the multivariate regression analysis, with a limited power due to the relatively small sample size. Heart failure may potentiate the development of AKI and vice versa [29]. First, several pathophysiological mechanisms activated in heart failure may affect the kidney: neurohormonal activation, venous congestion, inflammation, effects of pharmacologic therapy for heart failure. In turn, metabolic disturbances related to AKI (i.e., electrolyte imbalance, fluid overload) may be deleterious to the heart. Other interventions such as pharmacological therapies with SGLT2 have proven beneficial in patients with heart failure, reducing sudden cardiac death, lowering NT-proBNP levels, and preserving renal function [31,32]. Therefore, the observed improvement in oxygenation induced by HFNO during TAVR procedures may help break the vicious circle between heart and kidney failure. Moreover, obesity is a well-established risk factor for respiratory complications during sedation in relation to increased intra-abdominal pressure, augmented oxygen consumption from peripheral tissues, and airway collapsibility, with randomised controlled trials showing the benefits of HFNO on obese patients undergoing GI endoscopies [33,34]. However, in our study a high BMI was not a statistically significant risk factor for desaturation, which may be due to the relatively small number of obese patients recruited in the current study.

### Limitations

Some limitations must be acknowledged. Firstly, this trial is not powered for long-term clinical endpoints. Moreover, the relatively small sample size limits the power of the multivariate logistic regression analysis, increasing the risk of type II errors and possibly obscuring meaningful associations. Larger studies are guaranteed to confirm these findings. Secondly, this is a single-centre trial with a unique sedation protocol administered by anaesthesiologists. Whether the results may also apply to other sites/countries with different clinical practice (i.e., sedation administered by other professionals) can only be addressed in a multicentre trial. Third, given the nature of the trial, the use of HFNO cannot be blinded. However, endpoints were based on objective data not affected by blinding. Fourth, due to budget constraints we could not obtain the complete dataset of post-procedure NSE, NT-proBNP and hs-CTnI parameters. Therefore, the effects of HFNO on those biomarkers cannot be fully established from this trial. Fifth, the observed length of hospital stay may be longer than that of more experienced centres performing minimalist TAVR procedures. However, it is consistent with current reports on overall performance in different European countries with varying healthcare systems [35]. Lastly, despite the all-comers nature of the trial with broad inclusion criteria, only patients with Caucasian race were represented. Therefore, the results cannot apply to other races or ethnicities.

## 5. Conclusions

This trial demonstrated that HFNO provides a better oxygenation profile compared to standard oxygen therapy during sedation for TAVR procedures. In addition, the use of HFNO may help preserve renal function in this population. Further larger multicentre randomised controlled trials designed to determine the benefit of HFNO on reducing post-procedural complications after TAVR under sedation are warranted.

## Figures and Tables

**Table 1:** Baseline characteristics of patients receiving oxygen via HFNO or conventional nasal cannulas. Categorical variables are expressed as *n* (%). Continuous normally distributed variables are expressed as mean and standard deviation (SD) while continuous non-normal variables as median and (IQR).

Variable	H-g (*n* = 66)	S-g (*n* = 66)	SMD
**Age (years), mean (SD)**	80.6 (5.2)	81.5 (5.1)	0.175
**Female sex, n (%)**	25 (38)	25 (38)	<0.001
**Height (cm), mean (SD)**	164 (9.6)	163 (9.6)	0.061
**Weight (Kg), mean (SD)**	71.5 (12.0)	71.5 (11.9)	0.001
**BMI (Kg m^−2^), mean (SD)**	26.3 (3.9)	27.1 (4.7)	0.170
**Euroscore-II, median (IQR)**	2.7 (1.8–4.4)	3.2 (1.9–5.2)	0.247
**STS Score, median (IQR)**	3.6 (2.8–6.2)	4.7 (2.7–7.7)	0.291
**Hypertension, n (%)**	52 (79)	52 (79)	<0.001
**Dyslipidaemia, n (%)**	40 (61)	43 (65)	0.094
**Diabetes, n (%)**	19 (29)	17 (26)	0.068
**Atrial fibrillation, n (%)**	18 (27)	27 (41)	0.291
**COPD, n (%)**	13 (20)	10 (15)	0.120
**CKD, n (%)**	30 (46)	29 (44)	0.030
**Previous myocardial ischemia, n (%)**	15 (23)	15 (23)	<0.001
**Previous PCI, n (%)**	11 (17)	8 (12)	0.130
**PCI during TAVR, n (%)**	3 (5)	5 (8)	0.127
**pO_2_ (kPa), mean (SD)**	10.8 (1.9)	10.8 (1.7)	0.006
**pCO_2_ (kPa), mean (SD)**	4.8 (0.6)	4.7 (0.6)	0.211
**Aortic gradient (mmHg), mean (SD)**	47.7 (14.5)	46.7 (14.2)	0.073
**Left ventricle EF (%), mean (SD)**	56 (10.0)	55 (10.8)	0.155

SMD: standardised mean difference, SD: standard deviation; IQR: interquartile range; BMI: body mass index; STS: Society of thoracic surgeons; COPD: chronic obstructive pulmonary disease; CKD: chronic kidney disease; PCI: percutaneous coronary intervention; TAVR: transcatheter aortic valve replacement; EF: ejection fraction.

**Table 2:** Procedural characteristics including valve type and technical specifics in patients receiving oxygen via HFNO or traditional nasal cannulas.

Variable	H-g *n* = 64	S-g *n* = 61	*p*-Value
**BEV, n (%)**	17 (26.6)	14 (23.0)	0.253
**Sapien™**	13 (20.3)	8 (13.1)	
**Myval™**	4 (6.3)	6 (9.8)	
**SEV, n (%)**	47 (73.4)	47 (77.1)	0.253
**Evolut™**	26 (40.6)	25 (41.0)	
**Navitor™**	17 (26.6)	17 (27.9)	
**Acurate™**	4 (6.3)	5 (8.2)	
**Pre-dilatation, n (%)**	52 (81.3)	51 (83.6)	0.729
**Post-dilatation, n (%)**	8 (12.5)	8 (13.1)	0.918
**Intervention time, min, median (IQR)**	53 (40–134)	61 (43–142)	0.089
**Sedation time, min, median (IQR)**	67.5 (46–144)	76 (47–152)	0.066
**Amount of iodinated contrast (mL) median (IQR)**	130 (45–320)	135 (50–350)	0.485
**Length of stay, days, median (IQR)**	4 (1–28)	5 (2–85)	0.085

Categorical variables are expressed as n (%). Continuous are expressed as median and (IQR). BEV: balloon expandable valve; SEV: self-expandable valve; IQR: interquartile range; H-g: high-flow nasal oxygenation group; S-g: standard of care group.

**Table 3:** End-organ damage biomarkers measured before and 12 h after the procedure in patients receiving oxygen via HFNO or traditional nasal cannulas. Results expressed as median and (IQR).

Pre-TAVR Procedure				Post-TAVR Procedure (8–12 h After)		
	H-g *n* = 64	S-g *n* = 61	*p* -Value	H-g *n* = 64	S-g *n* = 61	*p* -Value
**Creatinine (mg dL^−1^)**	0.99 (0.55–5.94)	0.98 (0.57–2.57)	0.672	0.93 (0.41–6.41)	0.99 (0.43–4.16)	0.082
**Glomerular Filtration Rate (mL min^−1^ 1.73 m^−2^)**	67.13 (9.6–90)	57.94 (21.7–90)	0.532	71.73 (7.84–90)	59.66 (2.39–90)	0.029
NSE * **(ng mL^−1^)**	17 (10–41)	17 (9–36)	0.716	22 (11–44)	23 (12–40)	0.517
**hs-cTnI †** **(ng L^−1^)**	57.17 (3–1425)	83.98 (3–2858)	0.964	840.6 (5–9118)	945.3 (45–3746)	0.722
**NT-proBNP ‡ (pg mL^−1^)**	4082.8 (80–47,886)	3281.3 (60–18,012)	0.767	1778 (206–41,240)	1995 (64–31,917)	0.333

NSE: neuronal specific enolase; hs-cTnI: high sensitivity cardiac troponin I; NT-proBNP: N-terminal pro-B-type natriuretic peptide; H: high-flow nasal oxygenation; S: standard of care; TAVR: transcatheter aortic valve replacement. * NSE was obtained at baseline in 55 out of 64 (86%) in H-g and in 50 out of 61 (82%) patients in S-g; at post-TAVR, it was obtained in 40 out of 64 (63%) in H-g and in 37 out of 61 (61%) in S-g. † cTnI pre-TAVR was obtained at baseline in 62 out of 64 (97%) in H-g and in 56 out of 61 (92%) patients in S-g; at post-TAVR, it was obtained in 55 out of 64 (86%) in H-g and in 47 out of 61 (77%) in S-g. ‡ NT-proBNP was obtained at baseline 63 out of 64 (98%) patients in H-g and 60 out of 61 (98%) in S-g; at post-TAVR, it was procedural in 50 out of 64 (78%) in H-g and 47 out of 61 (77%) in S-g.

## Data Availability

The data presented in this study are available on request from the corresponding author.

## Supplementary Materials

The following supporting information can be downloaded at https://www.mdpi.com/article/10.3390/jcm14238347/s1, Figure S1: Changes in PaO_2_ across three time points (baseline, 45 min, and postoperative) in both study groups. Figure S2: Changes in PaCO_2_ across three time points (baseline, 45 min, and postoperative) in both study groups. Figure S3: Changes in NT-proBNP across two time points (baseline and postoperative) in both study groups. Figure S4: Changes in hs-cTnI across two time points (baseline and postoperative) in both study groups. Figure S5: Changes in NSE across two time points (baseline and postoperative) in both study groups. Figure S6: Univariate logistic regression analysis of desaturation events in the ITT population. Table S1: Delta (postoperative minus baseline) analysis of laboratory determinations across H-g and S-g. Table S2: Multivariate logistic regression analysis for desaturation events in the ITT population.

## Trial Registration

ClinicalTrials.gov number: NCT05958537. Registered on 29 June 2023.
