# Supplementary material for: High-Flow Nasal Oxygenation During Sedation for Transcatheter Aortic Valve Replacement: The HIGH-OXY-TAVR Randomised–Controlled Trial
Source: J Clin Med. 2025 Nov 24;14(23):8347. doi: 10.3390/jcm14238347 (PMC12693371; doi:10.3390/jcm14238347)
Supplement: Supplementary file 1 [file jcm-14-08347-s001.zip › jcm-3998117-supplementary.pdf]

Supplemental Material

High-flow nasal oxygenation during sedation for transcatheter aortic valve replacement: the HIGH-OXY-TAVR randomised-controlled trial.

Marc Giménez-Milà; Antoni Manzano-Valls; Omar Abdul-Jawad; María José Arguis; Salvatore Brugaletta; Thiago Carnaval; M José Carretero; Eduardo Flores-Umanzor; Xavier Freixa; Cristina Ibañez; Stefano Italiano; Manuel López-Baamonde; Samira Martínez; Purificación Matute; Mireia Pozo; Ricard Navarro; Juan Manuel Perdomo; Ander Regueiro; Irene Rovira; Francisco Javier Vega; Sebastián Videla, Manel Sabaté.

Supplemental Table S1 2
Supplemental Table S2 3
Supplemental Figure S1 4
Supplemental Figure S2 5
Supplemental Figure S3 6
Supplemental Figure S4 7
Supplemental Figure S5 8
Supplemental Figure S6 9

Supplemental Table S1: Delta (postoperative minus baseline) analysis of laboratory determinations across H-g and S-g

Parameter	Delta (Δ) *†	p-value
**Creatinine (mg dL^{-1}), median (IQR)**		
S-g	0.00 (–0.08 to 0.15)	0.006 *
H-g	–0.04 (–0.19 to 0.02)	
**GFR (mL min^{-1} 1.73 m^{-2}), median (IQR)**		
S-g	0.00 (–6.44 to 2.96)	0.003 *
H-g	1.77 (–0.96 to 8.49)	
**NSE (ng mL^{-1}), median (IQR)**		
S-g	5.00 (2.50 to 7.50)	0.246
H-g	3.00 (0.00 to 8.00)	
**NT-proBNP (pg mL^{-1}), median (IQR)**		
S-g	90.00 (–231.25 to 616.50)	0.250
H-g	44.50 (–249.00 to 264.00)	
**hs-cTnI (ng L^{-1}), median (IQR)**		
S-g	817.50 (347.70 to 1,672.20)	0.836
H-g	803.30 (316.80 to 1,723.00)	
**PaO_2 (Kpa), median (IQR)**		
S-g	1.16 (–1.00 to 3.38)	0.816
H-g	0.85 (–0.59 to 4.15)	
**$PaCO_2$ (Kpa), mean (95% CI) †**		
S-g	0.14 (–0.02 to 0.29)	0.448
H-g	0.05 (–0.10 to 0.21)	

S-g: Standard of Care-oxygen group; H-g: High flow Nasal Oxygenation group; GFR: Glomerular filtration rate; NSE: Neurospecific enolase; NT-proBNP: N Terminal pro-B-type natriuretic peptide; hs-cTnI: high sensitivity cardiac Troponin I.

* Statistically significant

† This variable followed a parametric distribution and was reported as mean (95% CI).

*† Delta (Δ) for non-parametric variables (Creatinine, GFR, NSE, NT-proBNP, hs-cTnI, and PaO_2) represent the median of the differences between postoperative and baseline values. Delta (Δ) for the only parametric variable ($PaCO_2$) represent the mean change between postoperative and baseline values.

Supplemental Table S2: Multivariate logistic regression analysis for desaturation events in the ITT population

Predictor	Estimate	SE	z-value	p-value	OR	95% CI
Intercept	0.248	0.883	0.281	0.778	1.282	0.227 to 7.235
Group	−1.584	0.506	−3.131	0.002 *	0.205	0.076 to 0.553
Sex (Male vs. Female)	0.038	0.545	0.070	0.944	1.039	0.357 to 3.025
BMI >30	0.406	0.583	0.697	0.486	1.501	0.479 to 4.704
Cr >1	0.175	0.824	0.213	0.832	1.192	0.237 to 5.997
GFR <60	−1.263	0.898	−1.407	0.160	0.283	0.049 to 1.644
NSE >17	0.205	0.508	0.403	0.687	1.227	0.453 to 3.321
NT-proBNP >1200	1.038	0.549	1.890	0.059	2.822	0.962 to 8.280
hs-cTnI >14	0.204	0.552	0.369	0.712	1.226	0.416 to 3.617
PaO_2 >10.7	−0.627	0.484	−1.296	0.195	0.534	0.207 to 1.379
$PaCO_2$ >4.7	−0.746	0.530	−1.408	0.159	0.474	0.168 to 1.340

* Statistically significant.

SE: Standard Error; OR: Odds Ratio; Cr: Creatinine; GFR: Glomerular filtration rate; NSE: Neurospecific enolase; NT-proBNP: N Terminal pro-B-type natriuretic peptide; hs-cTnI: high sensitivity cardiac Troponin I

Supplemental Figure S1: Changes in PaO_2 across three time points (baseline, 45 minutes, and postoperative) in both study groups.

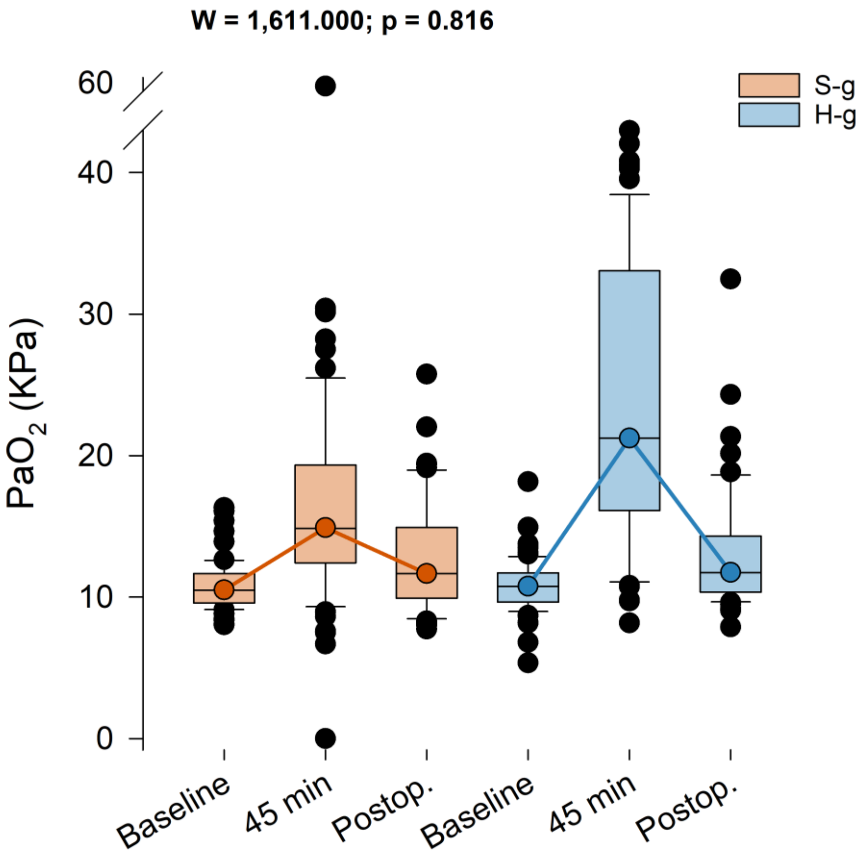

The p-value shown reflects the between-group comparison at 45 minutes (Wilcoxon rank-sum for PO_2, after normality check). Longitudinal trajectories across basal, 45 min, and post were analyzed using a GLMM (Gamma, log link) with random intercept and Type III tests for time×group interaction.

S-g: Standard of Care-oxygen group, H-g: High flow Nasal Oxygenation group; Postop.: Postoperative; W: Wilcoxon rank-sum test value.

Supplemental Figure S2: Changes in $PaCO_2$ across three time points (baseline, 45 minutes, and postoperative) in both study groups.

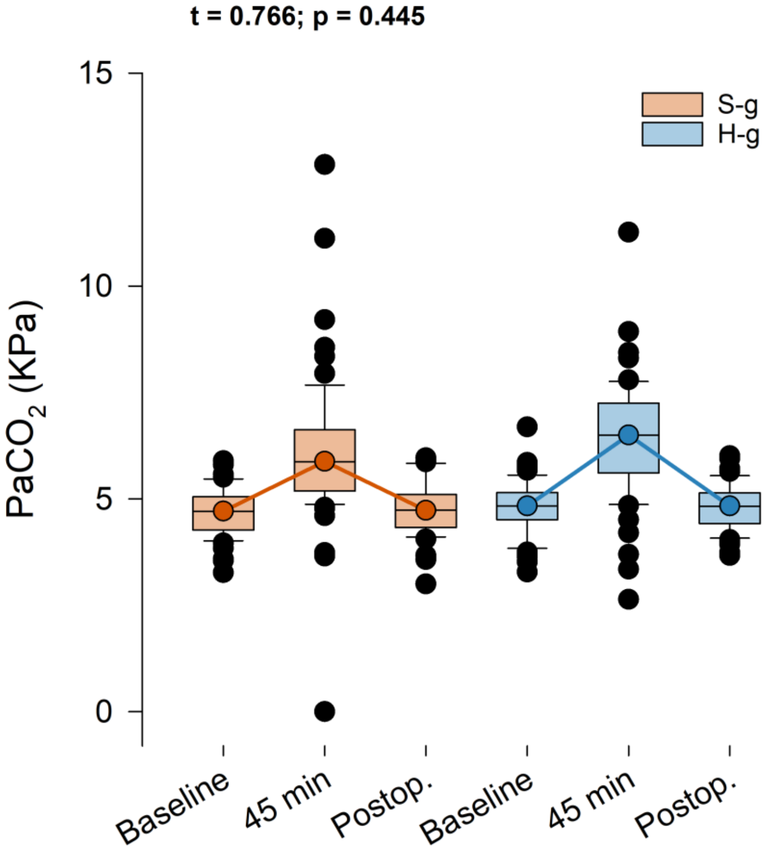

The p-value shown reflects the between-group comparison at 45 minutes (Welch's t-test for PCO_2 after normality check). Longitudinal trajectories across basal, 45 min, and post were analyzed using a GLMM (Gamma, log link) with random intercept and Type III tests for time×group interaction.

S-g: Standard of Care-oxygen group, H-g: High flow Nasal Oxygenation group; Postop.: Postoperative; t: Student's t test value.

Supplemental Figure S3: Changes in NT-proBNP across two time points (baseline and postoperative) in both study groups.

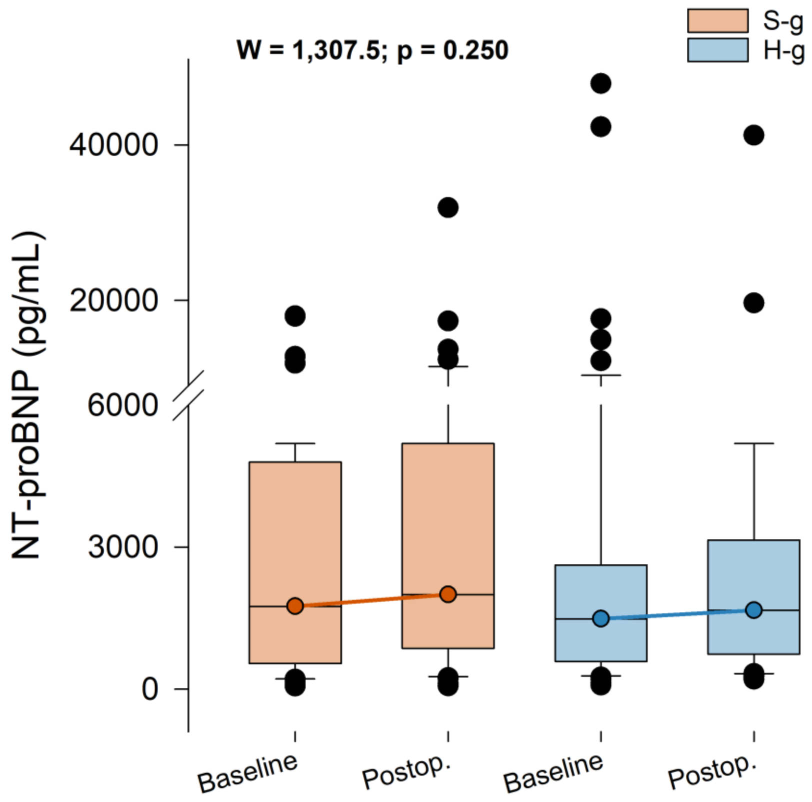

S-g: Standard of Care-oxygen group, H-g: High flow Nasal Oxygenation group; Postop.: Postoperative; W: Wilcoxon rank-sum test value. p-values correspond to comparison between post-procedure and basal accross groups with Wilcoxon rank-sum test.

Supplemental Figure S4: Changes in hs-cTnI across two time points (baseline and postoperative) in both study groups.

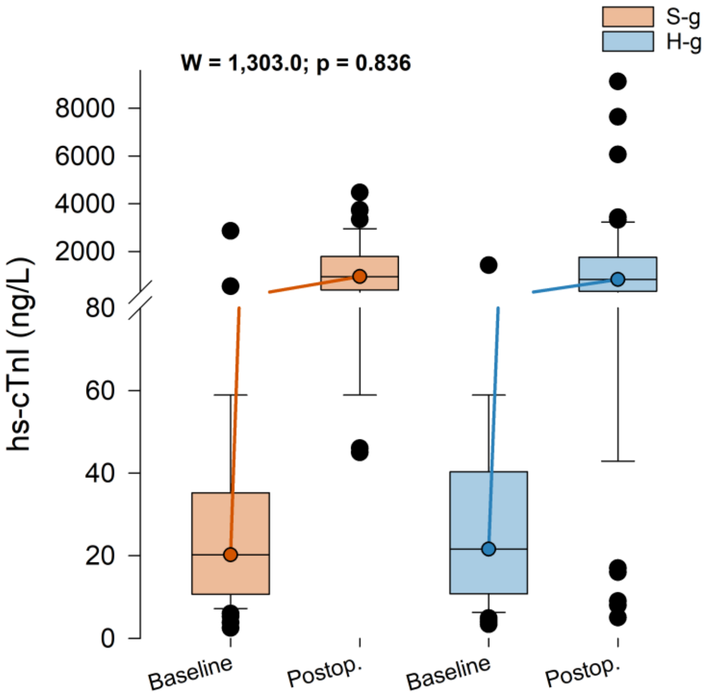

S-g: Standard of Care-oxygen group, H-g: High flow Nasal Oxygenation group; Postop.: Postoperative; W: Wilcoxon rank-sum test value. p-values correspond to comparison between post-procedure and basal accross groups with Wilcoxon rank-sum test.

Supplemental Figure S5: Changes in NSE across two time points (baseline and postoperative) in both study groups.

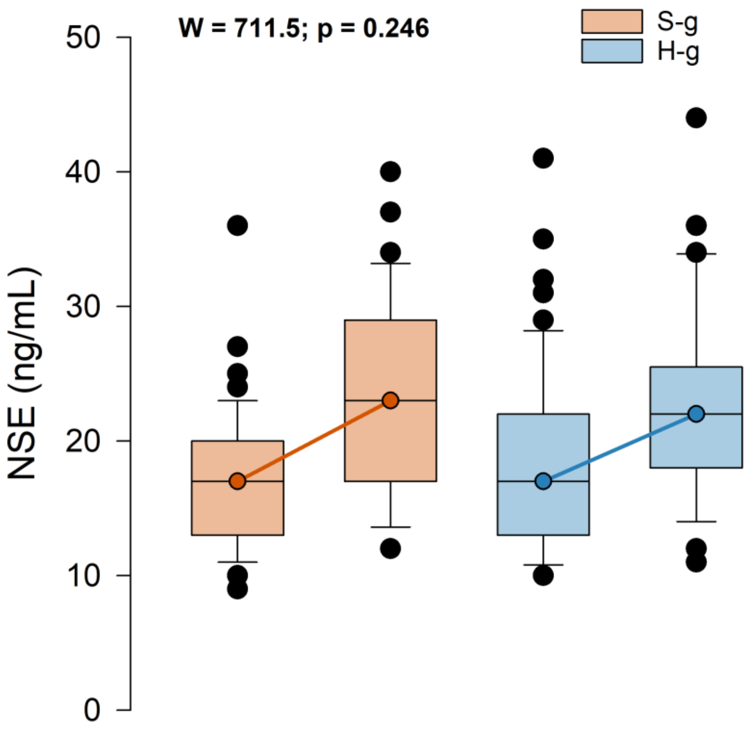

S-g: Standard of Care-oxygen group, H-g: High flow Nasal Oxygenation group; Postop.: Postoperative; W: Wilcoxon rank-sum test value. p-values correspond to comparison between post-procedure and basal accross groups with Wilcoxon rank-sum test.

Supplemental Figure S6: Univariate logistic regression analysis of desaturation events in the ITT population

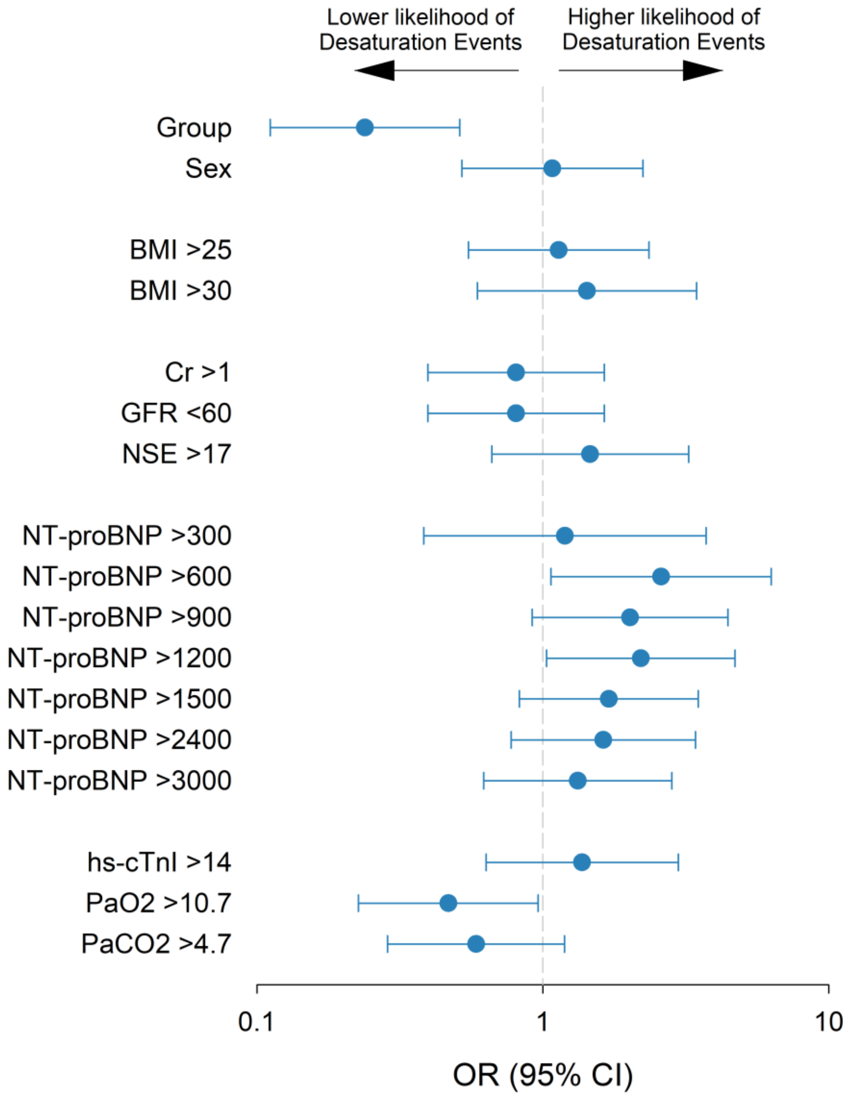

Group: Patients in the experimental group (H-g) had a significantly lower likelihood of experiencing desaturation events compared to those in the S-g.

Sex: Being male is associated with a slightly increased odds of experiencing a desaturation event compared to being female.

BMI: Body Mass Index; Cr: Creatinine; GFR: Glomerular filtration rate; NSE: Neurospecific enolase; NT-proBNP: N Terminal pro-B-type natriuretic peptide; hs-cTnI: high sensitivity cardiac Troponin I.
